# A Plant Distribution Shift: Temperature, Drought or Past Disturbance?

**DOI:** 10.1371/journal.pone.0031173

**Published:** 2012-02-10

**Authors:** Dylan W. Schwilk, Jon E. Keeley

**Affiliations:** 1 Biological Sciences, Texas Tech University, Lubbock, Texas, United States of America; 2 U.S. Geological Survey, Western Ecological Research Center, Sequoia-Kings Canyon Field Station, Three Rivers, California, United States of America; 3 Department of Ecology and Evolutionary Biology, University of California Los Angeles, Los Angeles, California,United States of America; University of Copenhagen, Denmark

## Abstract

Simple models of plant response to warming climates predict vegetation moving to cooler and/or wetter locations: in mountainous regions shifting upslope. However, species-specific responses to climate change are likely to be much more complex. We re-examined a recently reported vegetation shift in the Santa Rosa Mountains, California, to better understand the mechanisms behind the reported shift of a plant distribution upslope. We focused on five elevational zones near the center of the gradient that captured many of the reported shifts and which are dominated by fire-prone chaparral. Using growth rings, we determined that a major assumption of the previous work was wrong: past fire histories differed among elevations. To examine the potential effect that this difference might have on the reported upward shift, we focused on one species, *Ceanothus greggii*: a shrub that only recruits post-fire from a soil stored seedbank. For five elevations used in the prior study, we calculated time series of past per-capita mortality rates by counting growth rings on live and dead individuals. We tested three alternative hypotheses explaining the past patterns of mortality: 1) mortality increased over time consistent with climate warming, 2) mortality was correlated with drought indices, and 3) mortality peaked 40–50 years post fire at each site, consistent with self-thinning. We found that the sites were different ages since the last fire, and that the reported increase in the mean elevation of *C. greggii* was due to higher recent mortality at the lower elevations, which were younger sites. The time-series pattern of mortality was best explained by the self-thinning hypothesis and poorly explained by gradual warming or drought. At least for this species, the reported distribution shift appears to be an artifact of disturbance history and is not evidence of a climate warming effect.

## Introduction

The effects of predicted changes in precipitation and temperature may have complicated and potentially opposing effects on plants [1]–[3]. A recent study [4] reported changes in woody plant cover between 1977 and 2006 that indicated relatively rapid shifts in plant distribution along a southern California elevational gradient. The authors also noted that the region had experienced a temperature rise (0.6°C) during this period. This high-profile paper was the subject of a commentary by Breshears et al. [5] that unambiguously described the vegetation shift as resulting from “climate warming.” The fact that an observed shift is consistent with climate change, however, is a weak proof and other recent accounts of vegetation shifts have been the subject of controversy [2], [3], [6]. Consideration of the specific regeneration strategies of the species in [4], in fact, suggests that processes other than climate warming deserve more detailed examination.

In the semi-arid shrubland-dominated mountain ranges of California and other parts of the Southwest, plant recruitment and vegetation pattern are often linked to disturbance [7]. Past disturbance history and land use may covary with elevation, thus complicating efforts to explain apparent species elevation shifts [8]. The mean of a species' altitudinal distribution can shift due to mortality on the trailing edge, recruitment at or beyond the leading edge, growth on the leading edge, or some combination of these. Kelly and Goulden [4] recorded only live cover and presented no information on plant density and therefore no direct information on mortality or recruitment of individual plants. Their reported vegetation shift prompts the following questions: 1) was the shift observed between 1977 and 2006 due to mortality on the trailing edge, recruitment on the leading edge, or both? 2) how might the history of past fires in the mountain range have influenced their reported results? Many species that recruit following disturbance undergo extensive stand thinning as plants age and experience intense intra-specific competition [9], [10].

At least two of the nine species that contributed to the reported uphill elevation shift would not be expected to recruit seedlings in the absence of disturbance. For example, the chaparral shrub *Ceanothus greggii* has fire dependent germination; therefore, recruitment during the 30 years between surveys would be highly unlikely in the absence of fire. Similarly, *Pinus jeffreyi* often requires fire or other disturbance to provide suitable habitat for seedling recruitment. Most importantly, *C. greggii* is an “obligate seeder”: adults are killed by fire and regeneration occurs from a soil stored seedbank generating a pulse of even-aged recruits only in the first year after fire [11]. This life-history provides an opportunity to reconstruct past mortality rates. Live stems provide a reliable (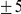
%) age since the last fire [12] and dead stems provide age at mortality.

We returned to the Deep Canyon Transect studied by Kelly and Goulden [4] and re-examined five elevational zones near the center of the gradient. These elevations are dominated by fire-prone chaparral and we tested a major assumption of the prior work that these sites had all experienced the same past fire history. Finding that this assumption was false, we examined what effect such differences might have on the reported upward shift by focusing on a single species, *Ceanothus greggii*. We sought to determine to what extent the apparent uphill distribution shift in *C. greggii* could be explained by climate warming, drought, or past disturbance history. We measured shrub densities as well as cover and we examined three alternative hypotheses for the pattern in *C. greggii* mortality over time, each represented by a generalized least squares model:

**H1** Mortality is associated with increasing temperatures

**H2** Mortality is associated with past episodic drought

**H3** Mortality patterns result from past fire history: intra-specific competition and self thinning lead to a mortality peak associated with time since fire

We examined four additional models representing combinations of these hypotheses: mortality is due to the combination of warming and drought (H1 & H2), mortality is due to warming and self thinning (H1 & H3), and mortality is due to self thinning and drought (H2 & H3). Finally we include a model that incorporated all three hypotheses (H1, H2 & H3).

## Results

Ring counts on live stem sections indicated that the sites were of different ages. Elevations 1340 m, 1463 m and 1585 m were 65 years old while the two highest elevation sites, 1707 m and 1829 m, were 91 years old. We found no evidence for shrub recruitment during the 1977–2006 period (live shrubs were even-aged within a site).

**Figure 1:**
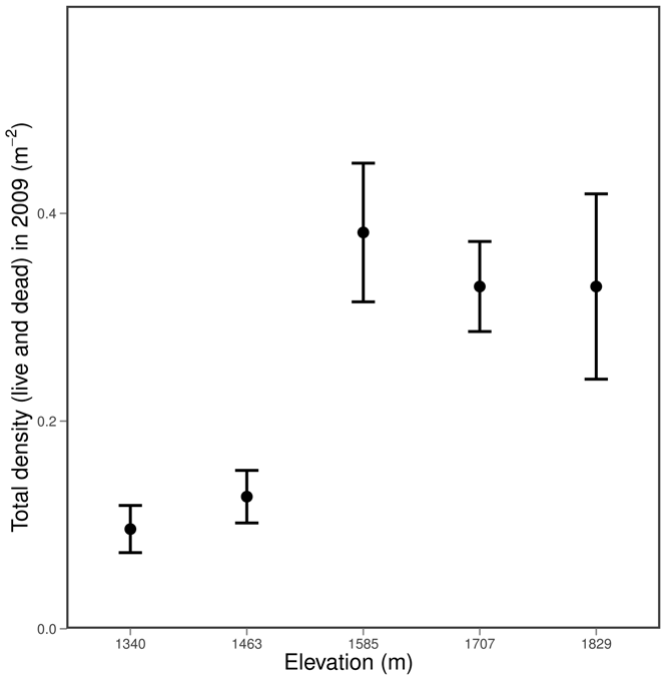
*C. greggii* density by elevation. Shown are mean densities 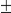 standard errors for 10 5×5 m plots per elevation.

**Figure 2:**
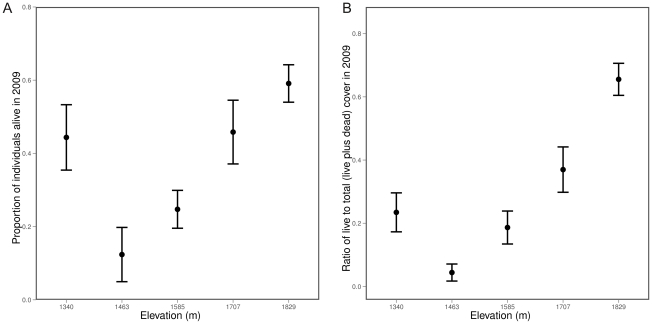
Live *C. greggii* cover and density relative to live and dead totals. Panel A shows the proportion of living stems to total number of stems (mean 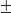 se). Panel B shows proportional contribution of live cover to total cover (mean 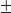 se).

Shrub live cover differed across the gradient with higher overall cover at the higher elevations (Table 1). Shrub densities and the ratio of live to total individuals varied across the five sites. Total densities (live and dead stems) tended to be highest at highest elevations (Fig. 1), but the pattern in the ratio of live to total stems and in live cover less clearly followed an elevational gradient (Fig. 2).

**Table 1:** Live cover of *C. greggii*.

Elevation	Mean cover (%)	SD
1340	4.3	4.7
1463	0.7	1.2
1585	6.4	6.5
1707	16.1	13.6
1829	19.7	14.7

Shown are means and standard deviations of 10 5×5 m plots at each elevation.

**Figure 3:**
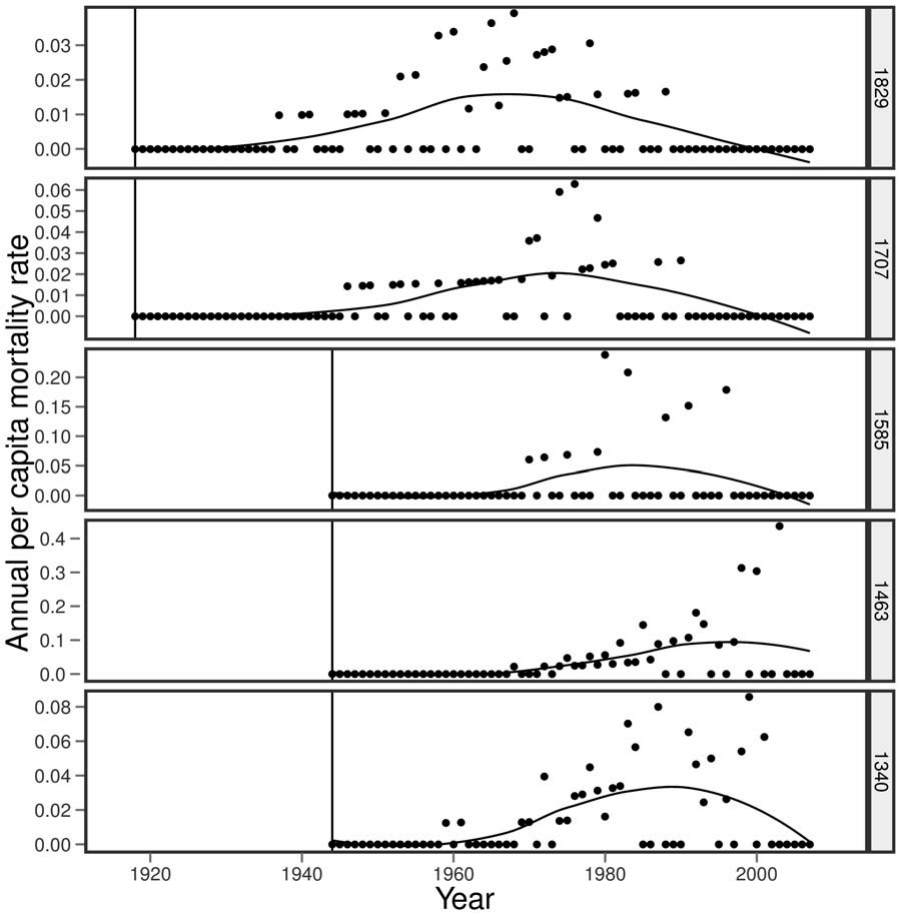
*C. greggii* per-capita mortality rates over time at five elevations. Vertical lines indicate year of stand establishment estimated from live stem ages. Fitted curves are loess curves [33] for overall illustration and estimation of year of peak mortality. The models that we considered are described in Table 2 and we used logit-transformed mortality rates. Stand age at peak mortality were estimated by loess smoothing (span = 0.75) from low to high elevation were: 45,50,40,56, and 48.

At each elevation, per-capita mortality showed a unimodal distribution with peaks in mortality 40–56 years following stand establishment (Fig. 3). The models that used age and drought alone as predictors fit the data poorly. The model representing the fire/self-thinning hypothesis (H3) received the highest model weight (41.4%) followed by the model with both a quadratic stand age term and a drought term (H2 & H3 model, weight of 24.1%). All of the remaining model weight was in the other two models that included the self-thinning hypothesis and quadratic age term (Table 2). These results indicate that mortality was best explained by fire history and stand self-thinning with a potential contribution by drought and perhaps temperature. Model ranking was unaffected by choice of the 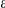 parameter in the empirical logit transform. Results of the Gaussian noise sensitivity analysis also supported our conclusion: coefficient estimates converged on the original values and AIC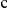-based model selection was stable across several orders of magnitude of noise (standard deviations of added error 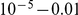).

**Table 2:** Model selection results based on generalized least squares mixed effects models of per-capita mortality rates.

	Hypothesis	Predictors	L.L.	K	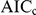	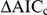	w 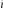
H3:	fire/self thinning	age, age 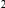	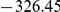	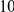	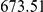	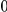	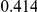
H2 & H3:	drought and fire/self thinning	age, age 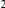 , PDSI	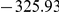	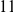	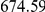	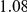	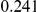
H1 & H3:	warming and fire/self-thinning	age, age 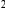 , temp	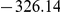	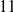	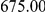	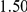	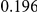
H1, H2 & H3:	warming, drought and fire/self-thinning	age, age 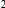 , PDSI, temp	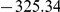	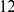	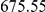	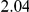	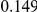
H1:	temperature	temp	−355.61	9	729.72	56.21	0.000
H1 & H2:	temperature and drought	PDSI, temp	−355.60	10	731.81	58.31	0.000
H2:	drought	PDSI	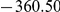	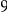	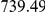	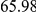	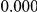

The seven models represent three alternative hypotheses and the four possible combinations of these hypotheses. Models are listed in order of 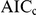 rank. Shown are the maximized log likelihoods (L.L), number of parameters (K), 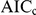, 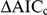, and the relative model weights (w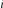). Predictors were three year running average summer temperature (temp), three year running average Palmer Drought Severity Index (PDSI), and stand age since fire (age).

## Discussion

These five elevations appear to have burned in at least two different past fires: one approximately 65 years ago and one 91 years ago. Kelly and Goulden [4] dismissed the possibility of fire history influencing the reported vegetation shift, but relied upon fire-history maps which are often inaccurate [13]. Kelly and Goulden [4] state: “This shift cannot be attributed to changes in air pollution or fire frequency…” That conclusion applies to all but the desert species and the method they used to rule out fire frequency for all species was based on fire history maps from the California Department of Forestry and Fire Protection's Fire and Resource Assessment Program (FRAP, http://frap.cdf.ca.gov/). Those maps indicate that the five elevations studied had the same fire history. Our study demonstrates those maps were wrong and Kelley and Goulden [4] were not studying sites with the same fire history. This incorrect assumption potentially affects all of the studied species, not just *C. greggii*.

Kelley and Goulden [4] insist that the reported shifts cannot be attributed to fire. They write: “Three considerations lead us to reject fire frequency as the main cause of plant redistribution. First, the degree of redistribution was independent of initial elevation (Table 1), whereas the degree of fire regime perturbation varied with elevation (20).” This statement is not justified; they are making a site specific observation and then ruling out fire based on broad regional generalizations. There is every reason to believe that their site could have a different fire history pattern with elevation and, indeed, our data show that to be the case. Furthermore, their conclusion is based on FRAP maps which are often inaccurate. A study of 250 sites in southern California [13] showed that 70 sites had no fire recorded on FRAP maps but ring counts indicated a fire during the period of record keeping. On the 180 sites with a recorded fire, the mapped age matched the true age (ring counts) on only 53%. On 47% of the sites where ages did not match, the true age was younger than the mapped age, indicating a missed fire. In short, true stand age was not mapped correctly by FRAP maps on 66% of sites. These are crude maps intended to give fire fighters broad landscape scale understanding of fuel loads and require verification when used as research tools.

Kelly and Goulden's second argument was regarding age since fire: “Second, most of the upper transect last burned in 1940 (20), which is relatively recent for Southern California montane forest (15). The fire frequency in the upper transect has not diverged markedly from the historic regime, and forested sections of the transect are not thought to have experienced large demographic changes from fire suppression (21).” This, too, is based on assumptions about the accuracy of FRAP maps and because the montane forests potentially burn in understory fires that are less likely to be detected, the possibility is high that those maps are even further off from the true stand age. The second sentence is contradicted by the source they cite. From Stephenson and Calcarone [14]: “The widespread and prolonged absence of fire is a concern in montane conifer forests. Fire-scar studies suggest that moderate intensity fires…historically occurred every fifteen to thirty years … However, over the last ninety years understory fires have been virtually eliminated from large areas… The result is denser stands and a dramatic increase in the number of understory trees…”

Finally, Kelly and Goulden [4] assert that past studies suggest that fire is unlikely to cause shifts in cover over elevation gradients: “… we are unaware of previous studies that have attributed a net altitudinal redistribution of vegetation (Fig. 3) to shifting fire frequency.” Studies do show, however, that different fire frequencies will produce different densities and cover. Therefore, if one observes elevational differences in fire frequency, then one should expect fire-induced changes in cover and density. We showed that their assumptions about fire were wrong and therefore their analysis falls short of what they contend. Our conclusion is further supported by the fact that we have demonstrated that, for one of the more abundant species, fire could have accounted for the patterns they observed.

Rather than indicating vegetation response to a warming climate, it appears that the mortality pattern in *C. greggii* results from different disturbance histories among the sites. The two higher elevation sites were older stands that had already peaked in mortality by the time of the 1977 sampling. These higher elevation sites then experienced proportionally less mortality during 1977–2006 compared to the three lower and younger sites and this resulted in an upward shift in the abundance-weighted mean elevation for *C. greggii*. Therefore, this apparent shift is best explained by past disturbance history and not temperature or drought alone. There is some evidence that drought and temperature may have had additional positive effects on mortality (Table 2). In these desert chaparral stands, mortality peaked approximately 50 years following stand establishment (Fig. 3). This pattern is consistent with the heavy self-thinning reported for other *Ceanothus* populations: *C. greggii* stands in San Diego County also peaked in mortality at around 50 years [10] and more productive *C. megacarpus* stands nearer the coast peaked in mortality at around 10 years [9].

Although we can only explore the past mortality rates for this species that has a life history allowing this analysis, the issue of past site history confounding reported shifts may be more general. In many mountain ranges, fire regime and past land use both co-vary with elevation [15], [16]. Past research has demonstrated range shifts due to disturbance [8] and biotic disturbances have been suggested as potential drivers of range shifts [17], [18]. Even apart from considerations of disturbance there are reasons to question the parsimony of the shifting upward assumption. For example, cold air drainage and inversions create altitudinal gradients in night time minimum temperatures that are often the reverse of daytime highs [19]. Under such conditions, plants responding to freezing temperatures [20] rather than to daytime highs and vapor pressure deficit may shift downward with warming climate. We argue that studies describing vegetation patterns consistent with climate change should include greater consideration of mechanism and include a thorough analysis of alternative hypotheses. This multiple hypotheses approach popularized by Chamberlin [21] is a good model for studies investigating climate change impacts [22].

## Materials and Methods

### Field methods and data collection

We resampled a subset of elevations along the Deep Canyon Transect in the Santa Rosa Mountains, California. We focused our attention on the five elevation bands at which *Ceanothus greggii* was recorded: 1340–1870 m [4], [23] (all sites on U.S. Forest Service land). Using the geographic coordinates in Kelly [19], we located the staked ends of each 400 m iso-contour transect. At each end and at every 100 m along this transect, we placed two 5×5 m plots: one on the uphill side of the transect and extending east and one on the downhill side and extending west. In each plot we recorded live canopy cover by species for all woody plants, live density of each woody species, and numbers of dead *C. greggii*.

At each elevation, we chose 2–3 living *C. greggii* individuals and these were harvested in order to estimate stand age from growth rings. We selected individuals near, but not on, the iso-contour transect and spread sampling across the 400 m transect. Stems were cut just above the root flare, which often required excavating several centimeters of soil before cutting because erosion since seedling establishment had covered the lower stem. Stem sections were sanded smooth and rings were counted under a stereo microscope at 10× power [24]. Due to the irregular growth patterns of these shrubs it is not possible to cross date and derive an exact year. However, past work has shown by vetting these counts against known stand age that such estimates were within 1–2 years in stands 30 years of age [12], [25].

To estimate past rates of mortality, we randomly selected 30–50 dead *C. greggii* individuals at each site and harvested these in the same manner as the live plants. The tendency for stems to separate required that we tape the stems before cutting. The sections from dead stems were sanded and rings counted as for live stems. We collected and sanded 209 stems in total, 35 of which had missing centers and in these cases we used the number of rings as an estimate of age although this underestimates stem age.

We used climate data from the Western Regional Climate Center (http://www.wrcc.dri.edu/) for California divisions 6 and 7 (South Coast Drainage and Southeast Desert Basin). Our temperature time series was the 3-year running average summer temperature (June, July, August) for the combined geographical divisions. As a time series of past drought, we used the 3-year running average of annual Palmer Drought Severity Index (PDSI) [26]. We explored several running average values and different yearly summaries, but those listed proved the most informative. No specific permits were required for the described field studies.

### Analyses

We were able to use the ages of live individuals to estimate stand establishment year. Assuming that all individuals established in the same post-fire year [11], we then used the ring counts from dead stems to establish year of mortality for each dead individual sampled. Ring counts were conducted in random order with the counter blind to the entire study. To estimate past mortality rates on a per-capita basis at each elevation, we used the mean live∶dead ratio among all 10 vegetation plots at each elevation to calculate an original cohort size associated with each group of dead stems (e.i., we multiplied the number of dead plants sampled at each site by the ratio of dead plants to total plants at the site and use this resulting number as an estimate of the initial cohort to which these dead individuals belonged). Cohort size each year is the number of living plants left in this group. By subtracting individuals according to the year they died from this cohort, we were able to reconstruct a per-capita mortality rate for each site for each year since stand establishment.

To evaluate our three alternative hypotheses, we used a model selection framework [27] in which each hypothesis was represented by a single model and four additional models represented combined hypotheses (Table 2). We accounted for temporal auto-correlation by using generalized least squares models and specifying the correlation structure. Model fitting and evaluation were accomplished using the gls function of the nlme library for R [28]–[30] and model selection was based on Akaike's Information Criterion adjusted for sample size (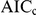) [27]. Each model included an autoregressive correlation structure of order 1 with year as the position variable and elevation (site) as a grouping variable (in some cases, nominal elevation dropped out a predictor when including it increased 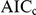). Similarly, for models that included temperature and or PDSI as a predictor, we tested if the site
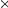
PDSI or site
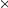
temperature interaction resulted in a lower 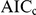, and if so, included the interaction term. This should eliminate bias against the temperature or drought hypotheses in the likely case that the strength of these effect varied with elevation. Models used the cohort size at each year as model weights (through the varExp function of the nlme package which predicted the variance as an exponential function of the cohort size) to allow for heteroscedasticity in mortality estimates. Annual mortality rates were transformed with an “empirical logit transform” [31] adding the smallest non-zero mortality rate (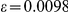 year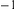) to both the numerator and denominator of the odds ratio as suggested by Warton and Hui [32] for modeling frameworks in which exact logistic regression is unavailable.

We compared five alternative models according to relative 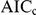 weights calculated from maximized log likelihoods [27]. Before model selection, we explored the importance of temporal auto-correlation and explored several variance structures (fixed, power relationship with cohort size, and exponential) separately for each model to ensure we did not bias our results against a hypothesis because a model had poor fit due to variance or correlation structure choice rather than our predictors of interest. The correlation structure and variance weight function we used were the best across all five models. To determine sensitivity to the empirical logit transform, we explored values of 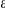 from 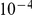 to 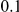.

In any given year, our estimate of per-capita mortality rate was necessarily coarse as it involved dividing a small number of aged dead stems by the estimated live cohort size for that year. We were concerned that the large number of constant values in the mortality time series (especially the many years with estimated zero mortality) might cause under- or over-estimation of coefficient confidence intervals and therefore influence model selection. To investigate this, we explored the behavior of the coefficient estimates and model rankings by incrementally adding increasing Gaussian noise (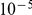 to 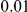) to the mortality rates before model fitting and recording coefficient estimates and relative model weights.

## References

[1] Wiens J, Stralberg D, Jongsomjit D, Howell C, Snyder M (2009). Niches, models, and climate change: Assessing the assumptions and uncertainties.. Proceedings of the National Academy of Sciences.

[2] Crimmins S, Dobrowski S, Greenberg J, Abatzoglou J, Mynsberge A (2011). Changes in climatic water balance drive downhill shifts in plant species optimum elevations.. Science.

[3] Stephenson N, Das A (2011). Comment on “Changes in climatic water balance drive downhill shifts in plant species' optimum elevations”.. Science.

[4] Kelly A, Goulden M (2008). Rapid shifts in plant distribution with recent climate change.. Proceedings of the National Academy of Sciences.

[5] Breshears D, Huxman T, Adams H, Zou C, Davison J (2008). Vegetation synchronously leans upslope as climate warms.. Proceedings of the National Academy of Sciences.

[6] Wolf A, Anderegg W (2011). Comment on “Changes in climatic water balance drive downhill shifts in plant species' optimum elevations”.. Science.

[7] Keeley J, Barbour M, Billings W (2000). Chaparral.. North American Terrestrial Vegetation, Cambridge University Press.

[8] Cannone N, Sgorbati S, Guglielmin M (2007). Unexpected impacts of climate change on alpine vegetation.. Frontiers in Ecology and the Environment.

[9] Schlesinger W, Gill D (1978). Demographic studies of the chaparral shrub, *Ceanothus megacarpus*, in the Santa Ynez Mountains, California.. Ecology.

[10] Zammit C, Zedler P (1993). Size structure and seed production in even-aged populations of *Cean- othus greggii* in mixed chaparral.. Journal of Ecology.

[11] Keeley JE (1991). Seed germination and life history syndromes in the California chaparral.. The Botanical Review.

[12] Keeley JE (1993). Utility of growth rings in the age determination of chaparral shrubs.. Madroño.

[13] Keeley J, Brennan T, Pfaff A (2008). Fire severity and ecosytem responses following crown fires in California shrublands.. Ecological Applications.

[14] Stephenson J, Calcarone G (1999). Southern California mountains and foothills assessment..

[15] Agee J (1991). Fire history along an elevational gradient in the Siskiyou Mountains, Oregon.. Northwest Science.

[16] Veblen TT, Kitzberger T, Donnegan J (2000). Climatic and human inuences on fire regimes in ponderosa pine forests in the Colorado Front Range.. Ecological Applications.

[17] Halloy S, Mark A (2003). Climate-change effects on alpine plant biodiversity: a New Zealand perspective on quantifying the threat.. Arctic, Antarctic, and Alpine Research.

[18] Cairns D, Moen J (2004). Herbivory inuences tree lines.. Journal of Ecology.

[19] Kelly A (2007). Shifts in the Deep Canyon Ecocline 1977–2007..

[20] Ewers FW, Lawson MC, Bowen TJ, Davis SD (2003). Freeze/thaw stress in *Ceanothus* of southern California chaparral.. Oecologia.

[21] Chamberlin T (1890, reprinted 1965). The method of multiple working hypotheses.. Journal of Geology.

[22] Platt J (1964). Strong inference.. Science.

[23] Zabriskie J (1977). Plants of Deep Canyon and the Central Coachella Valley, California..

[24] Keeley JE (1992). Demographic structure of California chaparral in the long-term absence of fire.. Journal of Vegetation Science.

[25] Coale T, Deveny A, Fox L (2011). Growth, re history, and browsing recorded in wood rings of shrubs in a mild temperate climate.. Ecology.

[26] Palmer WC (1965). Meteorological drought..

[27] Burnham K, Anderson D (2002). Model Selection and Multi-Model Inference: a Practical Information-Theoretic Approach..

[28] Pinheiro JC, Bates D (2000). Mixed-Effects Models in S and S-Plus..

[29] Pinheiro J, Bates D, DebRoy S, Sarkar D, R Development Core Team (2011). nlme: Linear and Nonlinear Mixed Effects Models..

[30] R Development Core Team (2011). R: A language and environment for statistical computing.. http://www.R-project.org.

[31] Collett D (2002). Modelling binary data..

[32] Warton D, Hui F (2011). The arcsine is asinine: the analysis of proportions in ecology.. Ecology.

[33] Cleveland W, Devlin S (1988). Locally weighted regression: an approach to regression analysis by local fitting.. Journal of the American Statistical Association.

